# Potential roles of stem cell marker genes in axon regeneration

**DOI:** 10.1038/s12276-020-00553-z

**Published:** 2021-01-14

**Authors:** Jinyoung Lee, Yongcheol Cho

**Affiliations:** Laboratory of Axon Regeneration & Degeneration, Department of Life Sciences, Korea University, Anam-ro 145, Seongbuk-gu, Seoul 02841 Republic of Korea

**Keywords:** Spinal cord injury, Peripheral nervous system, Molecular neuroscience

## Abstract

Axon regeneration is orchestrated by many genes that are differentially expressed in response to injury. Through a comparative analysis of gene expression profiling, injury-responsive genes that are potential targets for understanding the mechanisms underlying regeneration have been revealed. As the efficiency of axon regeneration in both the peripheral and central nervous systems can be manipulated, we suggest that identifying regeneration-associated genes is a promising approach for developing therapeutic applications in vivo. Here, we review the possible roles of stem cell marker- or stemness-related genes in axon regeneration to gain a better understanding of the regeneration mechanism and to identify targets that can enhance regenerative capacity.

## Nerve cells: activating genetic switches to repair injury

Understanding which genes are switched on/off in nerve cells in response to injury may help develop new treatments to repair nerves. Nerve cells can be cued to regenerate in both the central (brain and spinal cord) and peripheral nervous systems. Studying patterns of post-injury gene activation may allow therapeutic boosting of injury-responsive genes to cue nerve regrowth. Jinyoung Lee and Yongcheol Cho at Korea University, Seoul, South Korea have reviewed those genes identified as markers of stem cells (cells with high regenerative capacity) that are differentially regulated in nerve cells. They present several promising target genes for further study, noting that little is known of the mechanisms underlying regeneration or the interacting genetic pathways. These results point the way to identifying genetic targets to improve treatment for nerve damage.

## Introduction

Neuronal competence of axonal growth is regulated by transcriptional changes in response to injury^1^, and multiple genes responsible for axon regeneration have been identified by comparative gene expression profiling^2^. As the regenerative potential of injured neurons is distinctively regulated by both intrinsic and extrinsic factors^3–7^, differentially expressed genes under regenerative or nonregenerative conditions are considered potential targets for understanding the mechanism of axon regeneration that is orchestrated by injury-responsive genes^8–10^. Therefore, manipulating regeneration-associated genes (RAGs) is a potential method for developing approaches that promote regeneration. Diverse experimental designs have been utilized for comparative investigations of differential gene expression, revealing core genes that regulate regenerative capacity.

Tedeschi et al. reported three paradigms that produce the conditions for differential axonal outgrowth at different embryonic development stages, different regenerative stages of adult DRG neurons, and preconditioned adult DRG neurons^11^. They made a comprehensive list of genes that are differentially regulated based on the environmental conditions and are potential targets for manipulation that promotes axon regeneration. They identified *Cacna2d2* as a negative regulator of regeneration and presented a new method for promoting axon regeneration in vivo, as the pharmacological inhibition of *Cacna2d2* resulted in significantly improved regeneration in vivo; this work suggests that manipulating the expression of identified genes is a suitable strategy for promoting axon regeneration.

Stemness refers to the ability of a cell to perpetuate its lineage, produce differentiated daughter cells, and self-regulate its proliferation or regeneration^12^. Stemness is a manipulatable physiological characteristic, and the use of engineered pluripotent stem cells has ushered in a new era of regenerative medicine^13,14^. In addition, direct reprogramming technologies are emerging as novel approaches for treating neurological disorders and neural injury^15–17^. The elucidation of cellular stemness has resulted in the identification of genes that are specifically expressed in diverse types of stem cells. However, understanding how these genes regulate cellular stemness remains unclear. In addition, it remains unknown whether manipulating stem cell marker genes in nonstem cells might potentiate particular biological functions, such as regeneration and how they might be used to understand the mechanism by which stem cell marker genes mediate their effects.

**Fig. 1:**
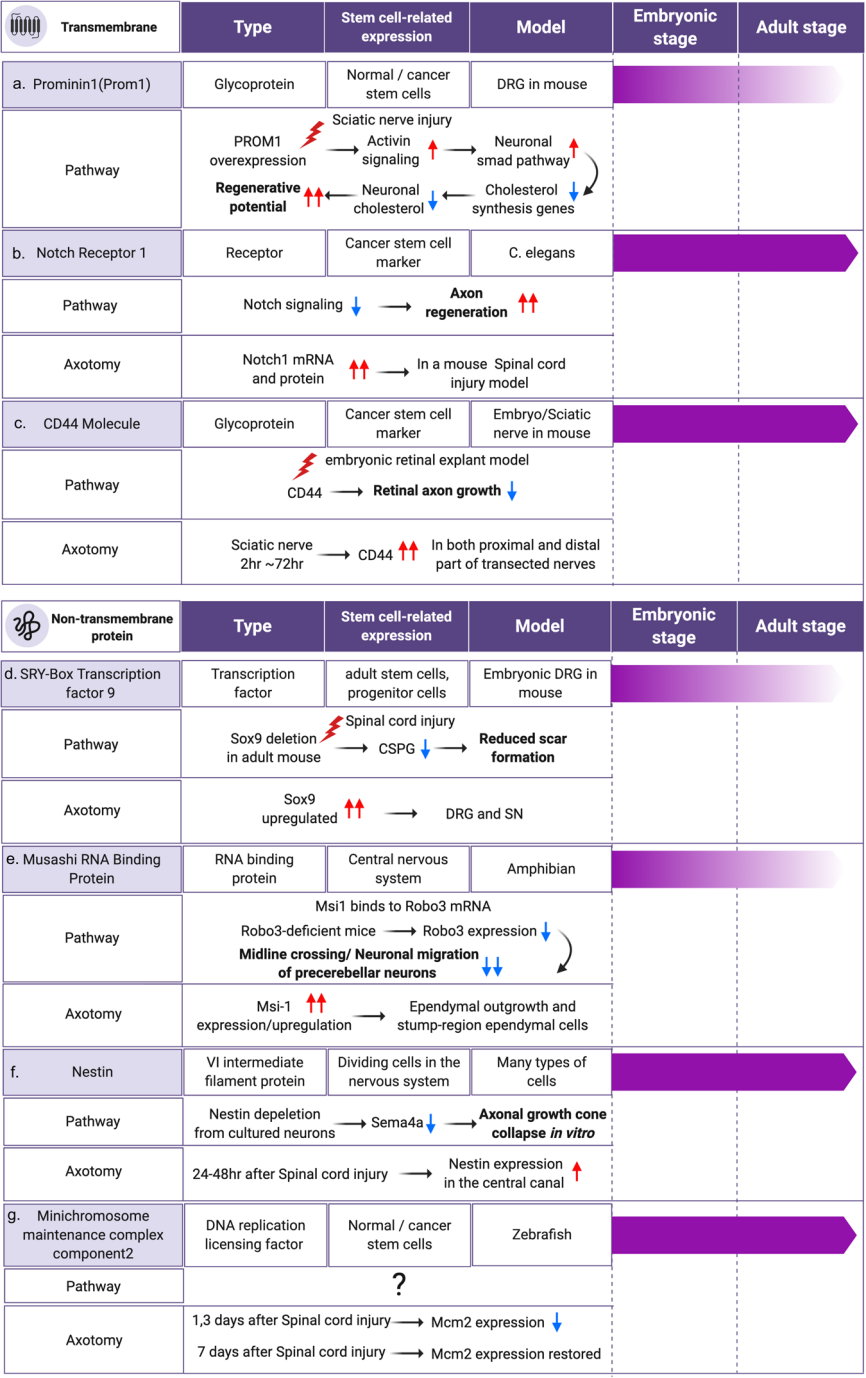
Illustration of stem cell marker genes that have potential roles in regulating axon regeneration. The schematic presentation describes the relative expression levels by developmental stage. All the references are included in the main text.

In relation to the potential function of stem cell marker genes and axon regeneration, research has recently shown that *Prom1*, a marker of stem cells, is expressed by dorsal root ganglion (DRG) neurons and is downregulated when neurons mature in adult mice^18^. *Prom1* overexpression in the DRG of adult mice in vivo resulted in the promotion of axon regeneration after sciatic nerve injury, implying that stem cell marker genes are potential candidates for developing new methods that enhance neuronal regenerative capacity. Here, we review neuronally expressed stem cell marker genes and their roles in axon regeneration and propose a new idea for establishing neuroregenerative applications related to cellular stemness (Fig. 1).

## SRY-box transcription factor 9 (Sox9)

*Sox9* encodes a transcription factor that is expressed in pluripotent, fetal, and adult stem and progenitor cells. *Sox9* expression is regulated by signal transduction pathways, such as the Sonic hedgehog pathway, Notch signaling, TGF-β pathway, and *Fgf9*-mediated signaling^19^. *Sox9* is downregulated in embryonic DRG tissues^11^. *Sox9* is expressed in astrocytes and ependymal cells in the neurogenic regions of adult human and mouse brains^20^. The expression of *Sox9* has been reported to be sustained across the Schwann cell lineage from E9 to E18.5 and at postnatal P7 and P65 in the mouse sciatic nerve^21^. In addition, non-myelinating Schwann cells also express *Sox9* after sciatic nerve injury. The expression of *Sox9* with characteristics of neural stem cells in the adult mouse brain was initially identified in the Bergmann glia population^22^. In embryonic DRG neurons, developmental downregulation was reported from E12 to E17 via RNA-Seq analysis^11^. *Sox9* is critical for the injury-responsive upregulation of genes associated with glial scar formation, which is a major barrier to axon regeneration in the CNS^23^; the genes associated with glial scar formation include those that encode chondroitin sulfate proteoglycans (CSPG). Tamoxifen-inducible *Sox9* deletion in adult mice has been reported to result in reduced CSPG production and small scar sizes in the spinal cord after injury, subsequently resulting in improved motor function recovery^23^ (Fig. 1d). These results indicate that inhibiting *Sox9* expression in glial cells is a potential approach for promoting functional recovery. Moreover, *Sox9* is upregulated in both DRG and sciatic nerves after axotomy^24,25^. Therefore, the roles of injury-upregulated *Sox9* expression must be investigated in PNS regeneration models.

## Musashi RNA-binding protein (Msi1)

*Msi1* is an RNA-binding protein (RBP) that regulates the translation of target mRNA and is known to be enriched in the CNS^26–28^. Musashi1 is required for the proliferation of neural progenitor cells, including CNS stem cells^29^; it binds to Robo3 to regulate posttranscription, a phenomenon that is essential for the midline crossing of pre-cerebellar neurons^30^ (Fig. 1e). In an investigation on *Msi1* in the ependymal cells of amphibian models wherein the levels of *Msi1* in the regeneration competent and noncompetent stages of *Xenopus* decreased, *Msi1* was found to be a robust marker of regeneration^31^. In addition, regeneration competent amphibians such as *Axolotl* and *Xenopus* exhibit different states of spinal cord ependymal cells. Axolotl express *Msi1* in embryonic and juvenile stages and not in adult stages; however, *Msi1* is upregulated after injury^31^. Moreover, in a functional screening, *C. elegans msi-1* was identified as critical for axon regrowth^32^. These reports indicate that Msi1-dependent cellular stemness may be a potential target for manipulation to promote axon regeneration. As RBPs participate in the posttranscriptional regulation of diverse mRNA stability, translational control, and localization^33^, Musashi-binding mRNAs that are associated with axon regeneration need to be identified for the elucidation of molecular mechanisms.

## Prominin1 (Prom1)

*Prom1*, a pentaspan membrane glycoprotein known to bind cholesterol, is used as a normal or cancer stem cell marker^34–36^ and is required for the formation of membrane protruding structures, such as tunneling nanotubes^37^. As genetic deletion of *Prom1* causes significant neural defects, such as retinal degeneration, a reduction in the number of neurons in the brain, and walking problems^38–41^, *Prom1* might have specific roles in neuronal tissues. In addition, transplanting prominin1-positive cells obtained from peripheral blood into an injured spinal cord enhanced angiogenesis, astrogliosis, and axon growth in damaged tissue, which led to the functional recovery of mice^42^. These results indicate that prominin1-regulated cellular physiology may be related to neural integrity and regeneration^43–45^. Comparative RNA-Seq analysis revealed that *Prom1* expression is developmentally downregulated in DRG neurons^11^, although it remains expressed in adult stages^18^. Furthermore, it was found that *Prom1* is critical for the differential regulation of a set of genes associated with cholesterol metabolism. *Prom1* overexpression promotes axon regeneration in vitro and in vivo and is mediated by the Smad pathway via the downregulation of cholesterol metabolism-related gene expression^18^. In addition, a reduction in cholesterol in DRG neurons significantly enhances axonal regeneration in vitro, suggesting that neuronal cholesterol level may be a potential target to manipulate the competence of axonal growth^18^. Although Jinyoung et al. showed that prominin1 interacts with ALK4, a receptor tyrosine kinase regulating Smad-TGF-β signaling^18^, the direct role of prominin1 in regulating the expression of a specific set of genes in DRG neurons is still unknown (Fig. 1a). The detailed mechanisms need to be further investigated to understand the functions of *Prom1* in neurons and nonneuronal cells, such as cancer stem cells.

## Nestin (Nes)

Nestin is encoded by *Nes* and is a type VI intermediate filament protein^46,47^. Owing to its high abundance in dividing cells in the nervous system, nestin is extensively used for identifying undifferentiated neuronal cells, although it is transiently expressed by many types of cells during developmental stages but is mostly absent as adulthood is approached. *Nes* is an effective marker of neural stem cells and progenitors, and nestin-positive cells engineered for therapeutic applications have been tested^48–51^. Interestingly, a subpopulation of embryonic cortical neurons has been found to transiently express nestin in its axons^50^. Moreover, nestin expression at the axonal ends of motile structures such as growth cones has been visualized. This observation indicates that nestin expression is not restricted to neuronal progenitor cells but is transiently regulated in neurons at specific developmental stages. In addition, as genetic alterations in neurofilament genes, including *NEFL*^52,53^, *NEF3*^52,53^, *NEFH*^54^, *DMN*^55,56^, *VIM*^57^, and *PRPH*^58^, have been reported in neurodegenerative diseases such as amyotrophic lateral sclerosis, Charcot-Marie-Tooth 2 and giant axonal neuropathy^59–61^, unidentified roles of Nestin in regeneration must be investigated (Fig. 1f).

## Minichromosome maintenance complex component 2 (Mcm)

Mcm2 is a protein required for genome replication and a key component of the prereplication complex^62^. Mcm2 is involved in the regulation of cell cycle progression in stem cells^63–65^ and is a marker of neuronal stem cells^66^. In gene expression profiling studies, Mcm2 was found to dynamically respond to injury after introducing spinal cord injury in zebrafish, as its expression was markedly reduced from 1 to 3 days after injury. However, the levels of Mcm2 returned to basal levels after 7 days and then significantly decreased^67^ (Fig. 1g). These findings suggested that critical factors regulating cell cycle progression, including Mcm2, are upregulated in response to spinal cord injury within a specific limited time window for regeneration. Interestingly, Mcm2 mRNA and Mcm7 protein, a member of the Mcm family located at the growth cone, were detected in mice^68^. These findings suggest that Mcm2 in regenerating axons may have specific functions that are not related to cell cycle progression.

## Notch receptor 1 (Notch1)

*Notch1* is required for the self-renewal of hematopoietic stem cells and inhibits differentiation^69,70^. Moreover, Notch 1 is a marker used to distinguish between normal and cancer stem cells^71,72^. In the nervous system, *Notch1* is required for neuronal and glial differentiation and activity-induced synaptic plasticity^73–75^. Single-neuron analysis of the regeneration of *C. elegans* indicated that Notch signaling negatively regulates axon regeneration and that inhibiting Notch signaling improves axon regeneration^76^. Notch acts as an intrinsic neuronal inhibitor of regeneration via canonical activation mechanisms regulated by ADAM metalloprotease. However, Notch-mediated inhibition has been reported to have a *DSL*-independent function, as *DSL/lag-2* did not inhibit but it promoted regeneration. In addition, the mechanism of injury-induced Notch activation is unclear owing to the lack of biochemical evidence describing Notch intracellular domain (NICD) levels upregulation after injury. However, overexpressing NICD inhibits regeneration via transcriptional functions. These results indicate that the target genes regulated by NICD, as well as the molecular mechanism activating Notch in response to injury, must be identified. Moreover, because the Notch pathway is regulated by multiple ligands^77^, injury-dependent Notch ligands must also be identified. Importantly, *Notch1* mRNA and protein were upregulated in an injury-responsive manner exclusively within neurons in a mouse spinal cord injury model^78–80^, suggesting that the role of injury-responsive *Notch1* upregulation needs to be investigated (Fig. 1b).

Direct evidence of NICD-mediated regulation of axon regeneration was reported by a study using a spinal cord injury model of sea lamprey^81^. Romaus-Sanjurjo et al. showed that GABOB a gamma-aminobutyric acid (GABA) analog and baclofen (a GABA receptor agonist) treatments promoted axon regeneration after spinal cord injury (SCI)^82^. Differential gene expression analysis was utilized to understand the molecular mechanism, and the results showed that *HESB* expression in the brainstem was significantly reduced by GABA or baclofen treatment 29 days after SCI^82^. *HESB* is one of the primary target genes of the Notch signaling pathway and functions as an effector of NICD^83^, implying that NICD negatively regulates axon regeneration after spinal cord injury via its downstream targets, including *HES* genes. This research group also showed that gamma-secretase inhibitor PF-3804014 treatment reduced the expression of *HESB* in the brainstem and enhanced axon regeneration^83^. This recent result shows that *HESB* is a potential downstream effector of Notch pathway-mediated axon regeneration. In addition, the target genes regulated by HES in response to injury need to be identified to understand the molecular mechanisms of NICD-regulated axon regeneration. In addition, the proteins interacting with NICD need to be investigated in axon injury models to identify their unidentified roles in regulating axon regeneration.

## CD44 molecule (CD44)

*CD44* is a transmembrane glycoprotein regulating cell-cell interactions. *CD44* is a stem cell marker of many types of cancer, such as bladder, breast, colorectal, gastric, head and neck, liver, lung, ovarian, pancreatic, and prostate cancers^84^. *CD44* participates in diverse cellular processes, such as cell adhesion and cell migration, by interacting with the extracellular matrix. *CD44* mRNA and protein were detected in the adult rat brain, and they colocalize with neuronal markers and astrocytes and regulate the structures and functions of dendritic spines^85^. *CD44* is involved in the regulation of cytoskeletal reorganization in the nervous system via small GTPases^86^. *CD44* inhibits retinal axon growth in vitro in an embryonic retinal explant model^87^. The role of *CD44* in axonal regeneration is not yet understood, although *CD44* expression is upregulated by axonal injury in a c-jun-dependent manner^88^. Recent transcriptome data indicate that *CD44* is a DLK-dependent injury-responsive gene, as indicated by the genetic deletion of DLK in sensory neurons in mouse DRG impairing the upregulation of *CD44* mRNA^24^. Notably, *CD44* is highly upregulated in the sciatic nerve quickly, within 2 h of injury, and its levels are sustained for as many as 72 h^25^. Furthermore, identical marked injury-responsive upregulation of *CD44* in both the proximal and distal parts of severed mouse sciatic nerves has been observed, suggesting that the role of *CD44* in regenerating and degenerating nerves must be considered^25^ (Fig. 1c). Because *CD44* undergoes alternative splicing, which produces multiple isoforms that have different biological functions, the injury-specific upregulated isoforms must be determined to understand the biological functions of *CD44* in axon regeneration.

## Conclusion

The elucidation of the mechanism underlying axon regeneration has been accelerated by gene expression profiling. Different biological conditions result in differential efficiency of axon regeneration, which is dependent on both neuronal extrinsic and intrinsic factors^5,7,89–91^. To understand the molecular mechanism of axon regeneration and identify methods for application, injury-associated gene expression has been utilized. By identifying injury-responsive genes via comparative analysis, the identification of new regulators of axon regeneration has become more feasible. However, recognizing only injury-regulated genes is not enough to define potential candidate genes or pathways for promoting axon regeneration because the differential expression of particular genes from injured neurons in experimental models is not always associated with neuronal regenerative capacity. Rather, a large number of DEGs identified through comparative analysis may simply display the transcriptomic landscape of injured neurons, which implies that additional ideas need to be considered for finding the targets for manipulation. Moreover, because it is still unclear which biological properties need to be augmented to enhance regeneration, the specific cellular function to be manipulated needs to be determined when we utilize target genes identified via DEG analysis.

Here, we review a group of genes involved in cellular stemness as target genes for studying axon regeneration. A set of genes that are significantly upregulated in stem cells have been identified and categorized as stem cell markers. However, the molecular functions of the individual genes in this set are still under investigation to understand the roles of stemness regulation. By recognizing that a group of stem cell marker genes shows developmentally decreased levels of expression^11,18^, the question of whether the expression levels of stem cell marker genes are related to the regenerative potential of injured neurons in adult animals can be answered. In addition, recent studies have shown that stem cell marker genes or their protein products are related to neurodegenerative disorders such as Alzheimer’s disease. For example, strong immunoreactivity indicated by anti-Msi1 staining was found in neurofibrillary tangle-bearing neurons in the hippocampus of patients with Alzheimer’s disease (AD)^92^. Moreover, the Msi-1 protein was present in an oligomeric state in AD brains, forming aggregates^93^. In addition, Notch1 expression was significantly higher in the hippocampus of patients with amyotrophic lateral sclerosis (ALS), although the immunoreactivity of NICD was lower, suggesting that the Notch pathway is inactivated^94^. However, the Notch pathway was abnormally overactivated in the spinal cords of sporadic ALS and ALS model mice^95^, suggesting that aberrant Notch pathway activation contributes to ALS pathogenesis. These reports clearly show that the Notch pathway and its related effectors are potential targets for understanding axon regeneration and degeneration^96^. CD44 expression in astrocytes and microglia is also known to be associated with ALS progression in a mouse model^97^, suggesting that the molecular interplay between stem cell marker genes from the nervous system needs to be investigated.

As indicated above, stem cell-associated genes are known to be differentially expressed in injured neurons or to display functions that regulate axon regeneration. Therefore, stem cell marker genes are potential candidates to manipulate for determining axon regenerative capacity in vitro and in vivo and to find downstream effector molecules that regulate regeneration, similar to the study on the Notch pathway in axon regeneration^81^. Although more research needs to be performed to gain a full understanding of the molecular connections between cellular stemness, the expression of stem cell marker genes, and neuronal regenerative potential, stemness and its related genes are potential targets to study molecular mechanisms regulating axon regeneration. Manipulating the expression levels of stem cell-associated genes that are selected from comparative transcriptomic data is one way to initiate this research. As a number of stem cell marker genes are transmembrane proteins, including *Prom1*, *CD44*, and *Notch1*, understanding the molecular mechanisms of membrane-associated stem cell markers that may lead to therapeutic applications may be realized by identifying ligand-like molecules regulating axon regeneration via membrane proteins.
